# Self-Efficacy for Coping with Breast Cancer Treatment Among Spanish-Speaking Latinas

**DOI:** 10.1089/heq.2020.0152

**Published:** 2021-04-26

**Authors:** Liliana Chacón, Jasmine Santoyo-Olsson, Cathy Samayoa, Alia Alhomsi, Anita L. Stewart, Carmen Ortiz, Cristian Escalera, Anna María Nápoles

**Affiliations:** ^1^ Division of General Internal Medicine, Department of Medicine, University of California San Francisco, San Francisco, California, USA.; ^2^ Department of Biology, SF BUILD Health Equity Lab, San Francisco State University, San Francisco, California, USA.; ^3^ Division of Intramural Research, National Institute on Minority Health and Health Disparities, National Institutes of Health, Bethesda, Maryland, USA.; ^4^ Department of Medicine, Center for Aging in Diverse Communities, Institute for Health & Aging, University of California San Francisco, San Francisco, California, USA.; ^5^ Círculo de Vida Cancer Support and Resource Center, San Francisco, California, USA.

**Keywords:** Latina/Hispanic, breast cancer treatment self-efficacy, patient engagement, spiritual wellbeing

## Abstract

**Background:** Cancer-related self-efficacy, a multidimensional construct, is the confidence that one can overcome challenges associated with cancer and its treatment; higher levels have been associated with better psychosocial outcomes of breast cancer survivors. Little is known about factors that influence it among Latina breast cancer survivors.

**Purpose:** Assess associations of several aspects of health care processes and of spirituality with self-efficacy for coping with breast cancer treatment among primarily Spanish-speaking Latina breast cancer survivors.

**Methods:** We analyzed baseline data from a randomized controlled trial of a cognitive–behavioral stress management intervention that enrolled 151 Spanish-speaking Latinas within 1 year of breast cancer diagnosis. Multivariate linear regression models examined associations of health care processes (quality of breast cancer care and information, participating in medical care, difficulty engaging with doctors) and spirituality (meaning/peace, faith, acceptance) with self-efficacy for coping with breast cancer treatment.

**Results:** Mean age was 51 (standard deviation [SD]=11), 66% completed high school or less, and most reported financial hardship in the past year (78%). Average time since diagnosis was 3.8 months (SD=2.7). In bivariate analyses, all six determinants were significantly associated with self-efficacy for coping with breast cancer treatment; participating in medical care (*B*=0.56, *p*<0.001) and having a sense of meaning/peace (*B*=0.76; *p*<0.001) were independently associated, controlling for sociodemographic and treatment characteristics.

**Discussion:** Interventions that promote participation in treatment decisions and sense of meaning and peace could improve confidence in coping with breast cancer treatment, and potentially quality of life, among Latinas living with breast cancer (Trial Registration Number: NCT01383174 [ClinicalTrials.gov]).

## Background

Breast cancer is the most common cancer in U.S. women; in 2020, an estimated 276,480 new cases of invasive breast cancer will be diagnosed among U.S. women.^1^ Approximately 24,000 of these cases are estimated to occur among Latinas.^2^

A breast cancer diagnosis can produce stress, anxiety, depression, and a sense of loss of control.^3,4^ Spanish-speaking breast cancer survivors report intense fear, powerlessness, and loss of control over their lives as a result of their illness.^5^ Latina breast cancer survivors are at higher risk of psychosocial morbidity and worse health-related quality of life than non-Latina women with breast cancer.^6^ These feelings intensify when deciding on and undergoing complex breast cancer treatments, such as surgery, radiation therapy, chemotherapy, and hormonal therapies.^3^ When initiating a chemotherapy regimen, 52% of Spanish-speaking Latinas reported clinically significant anxiety, and 27% reported depressive symptoms, reflecting high levels of emotional distress.^7^ Spanish-speaking Latinas undergoing treatment for breast cancer have higher rates of anxiety, depression, fatigue, pain, and fear of cancer recurrence and death than non-Latina women.^8,9^ Less than optimal quality of health care and aspects of spirituality have been implicated in these cancer-related psychosocial disparities.

Receipt of poorer quality of health care among Latinas could influence their confidence in the ability to manage cancer treatment. Latinas with breast cancer tend to experience poorer quality of care,^10^ less informational support from physicians,^11^ less patient-centered decision making, and multiple vulnerabilities that confer an elevated risk of loss of control over the cancer experience, poorer breast cancer-specific quality of life, and greater emotional distress.^12,13^ Limited information about cancer and its treatment resulted in increased fear and distress among Latina breast cancer survivors.^10,14,15^

Furthermore, limited English proficiency (LEP) among patients results in less effective communication with clinicians and limited involvement in cancer treatment decision making, factors that are associated independently with poorer outcomes of care.^10,16^ Relative to fluent English speakers or LEP patients seen by language-concordant health care providers, LEP patients seen by language-discordant providers report more problems understanding their health conditions, decreased satisfaction with health care, much less communication with their health care providers, and worse outcomes.^17,18^ A lack of accessible information about their diagnosis and treatment for Spanish-speaking cancer patients results in not fully understanding their diagnosis and treatment.^5^

Various cultural factors may also affect Latina breast cancer survivors' sense of their ability to manage their cancer treatment. Cancer fatalism, the belief that death is inevitable after a cancer diagnosis, is negatively associated with emotional wellbeing among Latinas with breast cancer.^19^ The literature tends to portray fatalism as a maladaptive coping behavior, implying a cultural deficiency (giving up).^8^ However, positive cultural scripts of spirituality (including nonreligious and religious components) and acceptance and their relationships with cancer self-efficacy have not received equal attention in studies among Latinas with breast cancer. Spirituality includes aspects related to acceptance of one's fate, religious beliefs, and prayer. Among Latinas, spirituality is an important coping strategy.^7,20–22^ Specifically, religious beliefs and prayer serve as sources of comfort and can aid in accepting cancer, which can reduce distress and improve quality of life.^23,24^ Acceptance of breast cancer is associated with better emotional wellbeing among Latina breast cancer survivors.^8^ Studies of spirituality among Latinas focus on religious beliefs; aspects of spirituality that are not directly related to faith, for example, sense of peace and meaning in life, have received less attention in Latina breast cancer survivors.

Cancer-related self-efficacy could be an important mechanism (mediator) of the effects of health care processes and spirituality on Latina breast cancer survivors' psychosocial outcomes. Self-efficacy is the confidence in one's ability to perform a behavior or achieve a desired outcome.^25^ Cancer-related self-efficacy focuses on the confidence that one can overcome various challenges of having cancer and undergoing treatment, such as managing symptoms and interacting with physicians.^26^ Low-income minority women with breast cancer with a greater sense of self-efficacy in interacting with physicians during diagnosis and treatment reported better quality of life.^27^ Among Latinos with breast or prostate cancer, higher levels of self-efficacy for managing patient/provider communication were associated with breast cancer-specific quality of life, less severe treatment side effects, and better emotional wellbeing.^28^ Also, in Latinas with breast cancer, higher levels of cancer-related self-efficacy related to the ability to get information, participate in care, and maintain a positive attitude were associated with less breast cancer symptom burden and cancer-specific distress.^26^

Despite their elevated risk of psychosocial distress, we have a poor understanding of how health care processes and spirituality affect cancer-related self-efficacy among Latina breast cancer survivors. In this study, we examined the extent to which health care processes (quality of breast cancer care and information, participating in medical care, and difficulty engaging with doctors) and spirituality (meaning/peace, faith, and acceptance) are associated with self-efficacy for coping with breast cancer treatment among Spanish-speaking Latina breast cancer survivors. If we can identify those factors that are associated with the degree of confidence in managing cancer treatment among these vulnerable women, such knowledge could inform the nature of psychosocial interventions for this group.

## Methods

### Study design

This study is a secondary analysis of baseline data from a randomized controlled trial (RCT) that tested the effectiveness of a cognitive–behavioral stress management program to reduce stress and improve health-related quality of life among Spanish-speaking Latinas living with breast cancer.^29,30^

The original RCT study was conducted in San Francisco Bay Area (Alameda, Contra Costa, San Francisco, San Mateo, and Santa Clara counties) settings that included cancer support agencies and public health system hospitals and clinics. Written informed consent was obtained from participants. The University of California San Francisco (UCSF) and San Mateo Medical Center Institutional Review Boards (IRBs) approved the protocol. The UCSF IRB was the IRB of record for the cancer agencies.

### Participants

The sample consisted of 151 women enrolled in the RCT between February 2011 and November 2013. Eligibility included self-identified as Latina and diagnosed with nonmetastatic breast cancer within the past year; 47% of participants had been diagnosed within the prior 3 months, and 85% were in the active treatment phase.

### Data collection

Recruiters were trained bilingual Latinas working with the community or clinical sites; they recruited, enrolled, and randomized participants and conducted in-person baseline assessments using structured surveys. The baseline assessment lasted ∼90 minutes and participants received $30. All participants completed the survey in Spanish. Further details on the study design and intervention are described elsewhere.^29,30^

### Measures

The dependent variable was self-efficacy for coping with breast cancer treatment. The six independent variables consisted of three aspects of health care processes related to women's breast cancer care and three cultural factors related to spirituality and acceptance.

We used available Spanish translations of measures when possible; otherwise, we performed translations using forward and backward translation methods, with team review and reconciliation. New and newly translated measures were pretested in Spanish through cognitive interviews with community residents meeting eligibility criteria (not included in the RCT study).

To measure *self-efficacy for coping with breast cancer treatment*, we used a 4-item scale adapted from the *coping and stress management* scale of the Brief Version of the Cancer Behavior Inventory (CBI-B) Version 2.0.^31^ Women rated their confidence that they can remain relaxed through treatment, manage nausea/vomiting, cope with physical changes, and not allow scary thoughts to upset them using a 1–9 scale with labels for the endpoints and midpoint, as follows: 1=not at all confident, 5=moderately confident, and 9=totally confident. We obtained permission to modify the original scale from the CBI developer. We dropped one item that asked about “maintaining work activity” because of very low item-scale correlations. The scale was then relabeled as *self-efficacy for coping with breast cancer treatment*, which reflected more precisely the item content. The scale is scored as the mean of nonmissing items, with a range of 1–9; higher scores indicate greater self-efficacy.

The three health care process factors related to breast cancer care were quality of breast cancer care and information, participating in medical care, and difficulty engaging with doctors. We developed a 2-item measure to assess perceived *quality of breast cancer care and information received about their breast cancer*. Women were asked to rate the care they received using a 5-point scale: 1=poor, 2=fair, 3=good, 4=very good, and 5=excellent. The 2-item scale was scored as the mean of nonmissing items, with a possible range of 1–5; higher scores indicate higher quality of care.

We used the *participating in medical care* scale from the CBI-B Version 2.0.^31^ The 2-item scale assesses women's confidence that they can participate actively in treatment decisions and ask doctors questions about their cancer care. The scale is scored as the mean of nonmissing items, with a range of 1=not at all confident to 9=totally confident; higher scores indicate greater participation. This measure assesses confidence in performing a specific behavior (asking questions and participating actively), which differs from measuring confidence to achieve an outcome (cope with treatment).

We developed a new 3-item scale assessing *difficulty engaging with doctors* based on our prior research with Spanish-speaking Latinos.^32^ Women rated the extent of difficulty they experienced asking doctors questions about cancer and cancer treatment, telling doctors what they want, and asking for an interpreter using a 4-point scale: 1=not at all difficult, 2=slightly difficult, 3=somewhat difficult, and 4=very difficult. The scale was scored as the mean of nonmissing items, with a possible range of 1–4; higher scores indicate greater difficulty engaging with doctors.

For the concept of spirituality, we used three measures. Two of these were from the Spanish version of the Functional Assessment of Chronic Illness Therapy (FACIT-Sp) Spiritual Well-Being instrument for cancer patients,^33^ consisting of two subscales assessing religious (*faith*) and nonreligious (*meaning/peace*) spiritual wellbeing. *Meaning/peace* is a 6-item scale assessing the extent to which respondents feel peaceful and have a sense of harmony, are able to find comfort within themselves, and have a reason to live. Based on psychometric analyses, we dropped two items that had low item-scale correlations, both of which were negatively worded (my life lacks meaning/purpose, trouble feeling peace of mind). The remaining items were positively stated. Cronbach's alpha was 0.81. *Faith* is a 4-item scale in which respondents rate the extent to which illness has strengthened their faith/spiritual beliefs, they find comfort and strength in faith/spiritual beliefs, and they know that whatever happens with illness, things will be okay. For both scales, respondents rated the extent to which each statement is true for them, with responses of: 0=not at all, 1=a little bit, 2=somewhat, 3=quite a bit, and 4=very much. Scores are the mean of nonmissing items (possible range 0–4); higher scores indicate greater levels of spirituality.

We derived our third measure of spirituality, *acceptance,* from a subset of items from the Benefit Finding scale originally developed by Tomich and Helgeson^34^ that was later adapted^35^ and translated into Spanish.^36^ Our 3-item *acceptance* scale assesses the extent to which having breast cancer has led to greater acceptance of things that cannot be changed (e.g., helped me take things as they come, adjust to things I cannot change) using a 5-point response scale: 0=not at all, 1=a little bit, 2=moderately, 3=quite a bit, and 4=extremely. Scores are the mean of nonmissing items, with a possible range of 0–4; higher scores indicate greater acceptance.

Self-reported descriptive characteristics included age, education, health insurance, employment status, financial hardship, marital status, national origin, English proficiency, presence of medical interpreter during breast cancer visits, and self-rated health. All breast cancer characteristics were verified through medical records, which included type of breast cancer, stage of breast cancer, type of surgery, and adjuvant therapy.

### Analysis

Descriptive statistics were used to characterize the sample. We examined psychometric properties of measures in this sample, aiming for item-scale correlations of >0.30 corrected for overlap, and internal consistency reliability >0.70.

Linear regression analyses were used to examine bivariate and multivariate associations of health care processes and spirituality with self-efficacy for coping with breast cancer treatment as the dependent variable. Covariates included age in years, education (sixth grade or less, seventh grade to high school diploma/GED, more than high school), and marital status (married or living with a partner, separated/divorced/widowed/never married). Breast cancer-related covariates extracted from medical records were stage of breast cancer (stage 0, I, II, or III), surgery type (lumpectomy, mastectomy), and treatment (both radiation and chemotherapy, radiation alone, chemotherapy alone or none). We report unstandardized regression parameter estimates (B) and standard errors.

## Results

Participants' mean age was 51 (standard deviation [SD]=11) (Table 1). The majority were of Mexican descent (68%) and about half were married or living with a partner (53%). Although most spoke English less than very well (88%), only 66% reported “often/always” having a professional medical interpreter during their breast cancer visits. The majority (66%) completed a high school level of education or less. Most had only public health insurance (82%) and experienced financial hardship in the past year (78%). Average time since diagnosis was 3.8 months (SD=2.7). Almost half received both adjuvant radiation and chemotherapy (40%).

**Table 1.** Baseline Characteristics of Spanish-Speaking Latina Breast Cancer Survivors: *Nuevo Amanecer* Study, San Francisco Bay Area, California, February 2011 to November 2013

Characteristic	Total sample, n=151
Age in years, mean (SD)	50.5 (10.9)
Education, *n* (%)	
Less than sixth grade	100 (66)
Sixth grade to less than high school	27 (18)
High school graduate	24 (16)
Health insurance, *n* (%)	
Private	21 (15)
Public	118 (82)
None	5 (3)
Employed full- or part-time, *n* (%)	26 (17)
Any financial hardship in past year, *n* (%)	115 (78)
Marital status, *n* (%)	
Married or living with a partner	80 (53)
Separated/divorced/widowed/never married	71 (47)
Country of birth (%)	
Mexico	102 (68)
Central America	35 (23)
South America	14 (9)
English proficiency, *n* (%)	
Very well/well	18 (12)
Not at all/poorly/fairly well	133 (88)
Professional medical interpreter present during breast cancer visits, *n* (%)	
Never	10 (8)
Rarely	8 (6)
Sometimes	27 (20)
Often	21 (16)
Always	66 (50)
Self-rated health, *n* (%)	
Poor/fair	96 (64)
Good/very good/excellent	53 (36)
Time since diagnosis, months, mean (SD)	3.8 (2.7)
Type of breast cancer, *n* (%)	
Ductal carcinoma *in situ*	40 (26)
Invasive	111 (74)
Surgery, *n* (%)	
Lumpectomy	84 (56)
Mastectomy	67 (44)
Adjuvant treatment, *n* (%)	
Both radiation and chemotherapy	60 (40)
Only radiation	42 (28)
Only chemotherapy	25 (17)
No treatment	24 (16)

SD, standard deviation.

All scales demonstrated item-scale correlations >0.40 and almost the entire possible range was observed for all scales (Table 2). Cronbach's alphas ranged from 0.68 for *participating in medical care* to 0.90 for *meaning/peace*.

**Table 2.** Descriptive Statistics, Internal-Consistency Reliability, and Item-Scale Correlations of Measures (*n*=151)

	Definition	Mean (SD)^a^	No. of items	Alpha	Range of item-scale correlations^b^	Possible range	Observed range
Dependent variable							
Self-efficacy for coping with breast cancer treatment	Confidence can manage nausea/vomiting, cope with physical changes, remain relaxed during treatment, control negative thoughts	6.36 (1.99)	4	0.76	0.51–0.67	1–9	1.25–9
Health care processes							
Quality of breast cancer care and information	Rating of overall medical care for breast cancer and information they have received about their breast cancer	4.06 (0.96)	2	0.86	0.75	1–5	1–5
Participating in medical care	Confidence they can actively participate in treatment decisions and ask doctors questions	7.60 (1.69)	2	0.68	0.52	1–9	2–9
Difficulty engaging doctors	Extent of difficulty asking questions about cancer and its treatment, telling doctors what they want, asking for interpreter	1.80 (0.83)	3	0.82	0.54–0.76	1–4	1–4
Spirituality							
Meaning/peace	Feels peaceful, has a sense of harmony, able to find comfort within self, has reason to live	2.96 (0.69)	6	0.81	0.45–0.73	0–4	1–4
Faith	Finds comfort and strength in faith/spiritual beliefs, illness has strengthened faith, knows that whatever happens with illness, things will be okay	3.43 (0.71)	4	0.90	0.67–0.89	0–4	0.25–4
Acceptance	Having breast cancer led to more acceptance of things that cannot be changed	2.59 (0.95)	3	0.89	0.74–0.85	0–4	0–4

^a^ Higher scores indicate more of the labeled construct.

^b^ Item scale correlations corrected for overlap.

In bivariate models, all three health care processes (*quality of breast cancer care and information, participating in medical care*, and *difficulty engaging with doctors*) and all three spirituality factors (*meaning/peace, faith*, and *acceptance*) were significantly associated with s*elf-efficacy for coping with breast cancer treatment* at *p*<0.001 (Table 3).

**Table 3.** Association of Health Care Processes and Spirituality with Self-Efficacy for Coping with Breast Cancer Treatment, *Nuevo Amanecer* Study, San Francisco Bay Area, California, February 2011 to November 2013

	Bivariate, B (SE); p-value	Multivariate,^a^ B (SE); p-value
Health care processes		
Quality of breast cancer care and information	0.62 (0.16); <0.001	0.12 (0.14); 0.40
Participating in medical care	0.72 (0.08); <0.001	0.56 (0.09); <0.001
Difficulty engaging doctors	−0.59 (0.19); <0.001	−0.17 (0.16); 0.28
Spirituality		
Meaning/peace	1.22 (0.21); <0.001	0.76 (0.24); <0.001
Faith	0.95 (0.22); <0.001	0.06 (0.23); 0.78
Acceptance	0.84 (0.16); <0.001	0.20 (0.15); 0.20; *R*^2^=0.489

^a^ Controlling for age, education, marital status, breast cancer treatment, breast cancer stage at diagnosis, and type of breast cancer surgery.

SE, standard error.

In multivariate models, controlling for covariates, *participating in medical care* was the only health care process to remain significantly associated with *self-efficacy for coping with breast cancer treatment* (*B*=0.56; *p*<0.001) and *meaning/peace* was the only aspect of spirituality that remained significantly associated with self-efficacy (*B*=0.76; *p*<0.001) (Table 3). The final model explained 49% of the variance in the outcome of *self-efficacy for coping with breast cancer treatment*.

## Discussion

This study sought to understand the extent to which health care processes and spirituality are associated with self-efficacy for coping with breast cancer treatment among vulnerable Latinas within the first year of diagnosis. Our findings that all of the quality of health care processes and aspects of spirituality were associated with cancer-related self-efficacy suggest the potential importance of these determinants. However, having a sense of meaning/peace and participating in medical care were the only ones that were independently associated with self-efficacy for coping with breast cancer treatment among this vulnerable sample of Latinas living with breast cancer.

Related to our finding of the importance of patient engagement in care, other studies among non-Latinas have shown that breast cancer survivors who are actively involved in treatment decision making are less likely to use cognitive avoidance coping and have better physiological adjustment than women who prefer passive involvement.^37^ Among Latinas with breast cancer-related symptoms, effective communication of cancer-related information has been linked to improved outcomes.^38^ For Latinas with LEP, being actively involved in their treatment decision making may be especially critical for enhancing their sense of control over their cancer treatment and in turn, improve outcomes of care.^10^

Our finding on having a sense of meaning and peace was positively associated with self-efficacy for managing breast cancer treatment is consistent with other studies of Latinos with cancer that showed that finding meaning and positive emotions helped them cope with a cancer diagnosis.^7,9,39^ Similarly, an intervention focused on meaning making-enhanced self-efficacy perceptions in women with breast cancer, although that study did not include Latinas.^40^ Our study adds to prior work by showing the link between finding meaning and an ability to control the cancer treatment experience specifically.

Study limitations include its cross-sectional nature, which precludes concluding causal relationships between health care processes, spiritual wellbeing, and cancer-related self-efficacy. Most participants in this study were of Mexican origin, therefore, our results may only be generalizable to this specific Latina subgroup. Participants also lived in predominately urban areas so results may not generalize to rural areas.

## Conclusion

In summary, our study found that participating in treatment decision making and asking questions are medical encounter processes that are associated with Latinas' confidence that they can cope with their breast cancer treatments. Focusing on cancer-related self-efficacy among this vulnerable group is crucial because they tend to experience poorer quality of care, less patient-centered decision making, and multiple vulnerabilities that result in a sense of helplessness. Furthermore, the literature has demonstrated that compared with their non-Latina counterparts, Latina breast cancer survivors experience psychosocial health disparities, for example, poorer breast cancer-specific quality of life and greater emotional distress.^3,6–8^ Moving toward achieving equitable cancer outcomes, our results support the need for greater attention to increasing the participation of Latinas in their cancer treatment decision making. Given that few studies have examined Spanish-speaking Latinas with breast cancer, our findings add important information that can be used to design programs that may improve the health outcomes of Latinas with cancer. For marginalized immigrant populations who face many structural barriers to resources and feel powerless when faced with a health crisis, increasing their sense of control in these situations could go far in improving their quality of life. Imbuing vulnerable Spanish-speaking Latinas with the confidence to manage the effects of their breast cancer treatments could begin to address these psychosocial health disparities.

## Abbreviations Used

CBI-B: Brief Version of the Cancer Behavior Inventory
IRB: Institutional Review Board
LEP: limited English proficiency
RCT: randomized controlled trial
SD: standard deviation
SE: standard error
UCSF: University of California San Francisco
